# Long-term exposure to PM_10_ and NO_2_ in relation to lung function and imaging phenotypes in a COPD cohort

**DOI:** 10.1186/s12931-020-01514-w

**Published:** 2020-09-23

**Authors:** Sung Ok Kwon, Seok Ho Hong, Young-Ji Han, So Hyeon Bak, Junghyun Kim, Mi Kyeong Lee, Stephanie J. London, Woo Jin Kim, Sun-Young Kim

**Affiliations:** 1 Biomedical Research Institutue, Kangwon National University Hospital, Chuncheon, South Korea; 2 Department of Internal Medicine and Environemntal Health Center, Kangwon National University, Chuncheon, South Korea; 3 Department of Environmental Science, Kangwon National University, Chuncheon, South Korea; 4 Department of Radiology, School of Medicine, Kangwon National University, Chuncheon, South Korea; 5 Division of Pulmonary and Critical Care Medicine, Department of Internal Medicine, National Medical Center, Seoul, South Korea; 6 National Institute of Environmental Health Sciences, National Institutes of Health, Department of Health and Human Services, Research Triangle Park, NC USA; 7 Department of Cancer Control and Population Health, Graduate School of Cancer Science and Policy, National Cancer Center, Goyang-si, Gyeonggi-do South Korea

**Keywords:** Air pollution, COPD, CT, Lung function, Traffic

## Abstract

**Background:**

Ambient air pollution can contribute to the development and exacerbation of COPD. However, the influence of air pollution on objective COPD phenotypes, especially from imaging, is not well studied. We investigated the influence of long-term exposure to air pollution on lung function and quantitative imaging measurements in a Korean cohort of participants with and without COPD diagnosis.

**Methods:**

Study participants (*N* = 457 including 296 COPD cases) were obtained from the COPD in Dusty Areas (CODA) cohort. Annual average concentrations of particulate matter less than or equal to 10 μm in diameter (PM_10_) and nitrogen dioxide (NO_2_) were estimated at the participants’ residential addresses using a spatial air pollution prediction model. All the participants underwent volumetric computerized tomography (CT) and spirometry measurements and completed survey questionnaires. We examined the associations of PM_10_ and NO_2_ with FVC, FEV_1_, emphysema index, and wall area percent, using linear regression models adjusting for age, gender, education, smoking, height, weight, and COPD medication.

**Results:**

The age of study participants averaged 71.7 years. An interquartile range difference in annual PM_10_ exposure of 4.4 μg/m^3^ was associated with 0.13 L lower FVC (95% confidence interval (CI), − 0.22- -0.05, *p* = 0.003). Emphysema index (mean = 6.36) was higher by 1.13 (95% CI, 0.25–2.02, *p* = 0.012) and wall area percent (mean = 68.8) was higher by 1.04 (95% CI, 0.27–1.80, *p* = 0.008). Associations with imaging phenotypes  were not observed with NO_2_.

**Conclusions:**

Long-term exposure to PM_10_ correlated with both lung function and COPD-relevant imaging phenotypes in a Korean cohort.

## Introduction

Air pollution is an important risk factor for the mortality and morbidity of cardiorespiratory diseases globally [1]. Global estimates of premature deaths and disability-adjusted life-years from COPD by air pollution are 0.86 and 16.8 million in 2015 [2]. Increased short-term exposure to ambient air pollution for a few days is associated with respiratory mortality and exacerbation of respiratory diseases leading to hospital admission [3–5]. Long-term exposure to ambient air pollution for years has been associated with reduced lung function and also can contribute to the development and exacerbation of COPD [6–9]. These studies focused on concentrations of traffic-related air pollutants such as particulate matter less than or equal to 10 or 2.5 μm in diameter (PM_10_ or PM_2.5_) and nitrogen dioxide (NO_2_). In recent years, more refined methods have been developed to adequately estimate individual-level air pollution concentrations at residential addresses [10].

Recent advances in computed tomography (CT) measurement lead to understanding of the clinical implications of emphysema severity and airway wall thickening. Emphysema is an important structural feature of COPD and is associated with adverse outcomes with or without COPD [11, 12]. Airway wall thickening measured by CT was associated with cigarette smoking and disease severity [13]. However, only few studies have examined the effects of air pollution on these imaging phenotypes so far [14–16]. Previous studies were performed in Western countries. Genetic factors and nature of the PM may differ across regions. Studies based on a well-designed cohort including COPD patients, diverse environmental exposure data, and imaging measures can clarify the effects of air pollution on imaging phenotypes as well as lung function [17].

The COPD in Dusty Areas (CODA) cohort in South Korea was constructed focusing on the people living near cement plants in Gangwon and Chungbuk provinces, South Korea [18–20] and employed a recently-developed air pollution prediction model for improved exposure assessment at the individual level [21]. We investigated the association between traffic-related air pollution and both lung function and quantitative imaging phenotypes including emphysema severity and airway measurements. Some of these results have been previously presented as an abstract [22].

## Methods

### Study population

A total of 504 subjects who resided in areas near cement plants were recruited in the CODA cohort between 2012 and 2017 in South Korea. We recruited participants from affected administrative districts that were selected by the National Institute of Environmental Research of Korea based on the distances and wind direction to cement plants. We mailed an invitation and then subsequently called each subject whose address was located within our pre-defined area of study. Subjects include those having or not having airflow limitations based on spirometry. The protocols of data collection in the CODA cohort were previously described in detail [23–25]. In brief, we obtained data on demographic characteristics, medical history, and environmental exposures from participant questionnaires.

### Spirometry and imaging procedures

Lung function was measured before and after administrating 400 μg of salbutamol using EasyOne (NDD, Zurich, Switzerland) and pulmonary function measures were selected according to ATS/ERS criteria [26]. We focused on FEV_1_ and FVC as the two lung function outcomes in this study. COPD status was defined as a post-bronchodilator FEV_1_/FVC less than 0.7 at baseline. CT measurements were obtained using a dual-source CT scanner (Somatom Definition, Siemens Healthcare, Forchheim, Germany) at full inspiration and expiration in the supine position. Emphysema index was calculated as the percentage of lung area below − 950 HU threshold, while wall area percent was defined as (100 x wall area/total bronchial area) to assess airway thickness and was measured near the origin of the right apical and left apicoposterior segmental bronchi using in-house software and the two measurements were averaged [25, 27, 28]. Functional small airway disease was calculated as a percentage of lung area between ≥ −950HU at inspiration and < −856HU at expiration after image co-registration of inspiratory and expiratory CT using an Aview® system (Coreline Soft Inc., Seoul, South Korea). Written informed consent was given by each participant. This study received ethical approval from the Kangwon National University Hospital IRB (KNUH 2012–06-007, clinical trial registration number KCT-0000552).

### Air pollution exposure assessment

Annual average concentrations of PM_10_ and NO_2_ at participants’ home addresses were estimated from a previously-developed air pollution prediction model. The details of this model have been described previously [21]. Based on the air quality monitoring data for 2010 in South Korea, this model estimated annual average concentrations at any location in South Korea using a universal kriging framework that consists of summary predictors of about 300 geographic variables and spatial correlation of air pollution concentrations. The cross-validated R^2^ values indicating the prediction ability of the model were 0.45 and 0.82 for PM_10_ and NO_2_, respectively. This model performance was comparable to those of national-scale prediction models in North America and Europe [29–31].

### Statistical analyses

To investigate the association of PM_10_ and NO_2_ with FEV_1_, FVC, emphysema, and wall area percent, we performed linear regression analysis adjusting for individual characteristics. Separate models were applied to each pair of two pollutants and four outcomes. We used two models to examine the sensitivity of our results to the progressively-added confounding variables. In model 1, we adjusted for age, gender, education, smoking, height, weight, occupation, and medication for COPD to our primary model. Smoking was identified as smoking status and smoking amount in pack-years. We analyzed job in 3 groups: cement worker (regular and higher dust exposure); farmer (less frequent and lower dust exposures), all other jobs (no dust exposure). Model 2 additionally included the calendar year of pulmonary function testing, and asthma history and COPD status were added in model 3. We presented the effect estimate for an interquartile increase (IQR) in each pollutant concentration to allow the comparison given the different scales of the two pollutants. We also performed subgroup analyses stratified by gender, the status of COPD, smoking, and overweight/obesity, and underwent statistical tests of interaction using product terms with PM_10_ or NO_2_. Smoking status was categorized to never vs. ever (combining former and current) smokers. Overweight/obesity was defined as a BMI ≥ 23 kg/m^2^, according to the World Health Organization Asia–Pacific criteria [32]. All statistical analyses were performed using SAS version 9.4 (SAS Institute Inc., Cary, NC). The *p* value < 0.05 was defined as indicating statistical significance.

## Results

### Characteristics of the CODA cohort participants

**Fig. 1:**
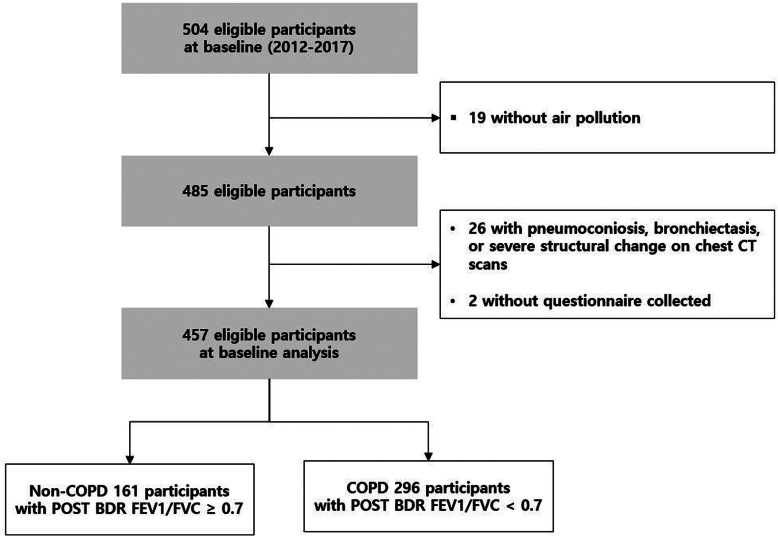
Flowchart for study participation in the COPD in Dusty Areas (CODA) cohort study

There were 457 participants included in our study. (Fig. 1) The mean age was 71.7 years and the mean BMI was 23.5 kg/m^2^. There were 165 never (36%), 194 former (43%), and 98 current smokers (21%). Among the participants, 170 subjects (38%) had an occupational history of a cement factory worker and 149 subjects had a history of a farmer. The average post-bronchodilator FEV_1_ and FVC were 1.96 and 3.02 L, respectively (Table 1). The average emphysema index was 6.36 and the mean wall area percent was 68.8%. Among all, 296 subjects (65%) were COPD patients and 161 subjects were non-COPD.

**Table 1:** Participant characteristics at baseline in the Korean CODA cohort (*n* = 457)

	All (*n* = 457)	Non-COPD (*n* = 161)	COPD (*n* = 296)
Gender			
Male	335 (73.3)	97 (60.2)	238 (80.4)
Female	122 (26.7)	64 (39.8)	58 (19.6)
Age	71.7 ± 7.3	70.8 ± 7.7	72.2 ± 7.1
44 ~ 59 yr	29 (6.3)	15 (9.3)	14 (4.7)
60 ~ 69 yr	113 (24.7)	42 (26.1)	71 (24.0)
70 ~ 79 yr	260 (56.9)	91 (56.5)	169 (57.1)
80 ~ 96 yr	55 (12.0)	13 (8.1)	42 (14.2)
Education			
< Elementary school	143 (32.0)	43 (27.7)	100 (34.2)
Elementary school	169 (37.8)	67 (43.2)	102 (34.9)
Middle school	65 (14.5)	23 (14.8)	42 (14.4)
≥ High school	70 (15.7)	22 (14.2)	48 (16.4)
Income (x10^4^won)			
≤ 49	280 (63.9)	95 (62.5)	185 (64.7)
50–99	70 (16.0)	22 (14.5)	48 (16.8)
≥ 100	88 (20.1)	35 (23.0)	53 (18.5)
Job			
Cement factory	170 (37.2)	55 (34.2)	115 (38.9)
farmer	149 (32.6)	62 (37.9)	87 (29.3)
Others	138 (30.2)	44 (27.3)	94 (31.8)
Smoking			
Never-smoker	165 (36.1)	87 (54.0)	78 (26.4)
Former-smoker	194 (42.5)	52 (32.3)	142 (48.0)
Current-smoker	98 (21.4)	22 (13.7)	76 (25.7)
Pack-years	17.6 ± 23.4	12.0 ± 18.5	20.6 ± 25.2
Height (cm)	159.4 ± 9.3	157.8 ± 10.3	160.3 ± 8.6
Weight (kg)	59.7 ± 10.4	60.0 ± 10.6	59.6 ± 10.3
BMI (kg/m^2^)	23.5 ± 3.2	24.0 ± 3.3	23.2 ± 3.2
< 23.0	207 (45.3)	64 (39.8)	143 (48.3)
23.0 ~ 24.9	106 (23.2)	40 (24.8)	66 (22.3)
≥ 25.0	144 (31.5)	57 (35.4)	87 (29.4)
History of COPD medications			
No	362 (79.2)	149 (92.5)	213 (72.0)
Yes	95 (20.8)	12 (7.5)	83 (28.0)
Asthma, history of disease			
No	376 (83.9)	136 (87.7)	240 (81.9)
Yes	72 (16.1)	19 (12.3)	53 (18.1)
Pulmonary outcome at baseline visit, Post BDR			
FVC, L	3.02 ± 0.81	2.88 ± 0.80	3.10 ± 0.81
FVC, % predicted	97.8 ± 19.1	96.9 ± 18.9	98.3 ± 19.3
FEV_1_, L	1.96 ± 0.60	2.19 ± 0.61	1.84 ± 0.56
FEV_1_, % predicted	87.3 ± 22.5	100.7 ± 21.1	80.0 ± 19.7
FEV_1_/FVC	0.65 ± 0.11	0.76 ± 0.05	0.59 ± 0.08
Emphysema index, *n* = 414	6.36 ± 6.66	3.35 ± 3.60	7.64 ± 7.23
Wall area %, *n* = 414	68.8 ± 5.2	67.5 ± 5.4	69.3 ± 5.0

Data are mean ± SD for continuous variables and n(%) for categorical variables

### Exposure to air pollution

The summary statistics of the individual-level air pollution concentrations are shown in Table 2. Annual average concentrations of PM_10_ and NO_2_ predicted at 457 CODA cohort participants’ homes in 2010 were 43.1 ± 2.9 μg/m^3^ was 13.6 ± 2.1 ppb, respectively. These were lower than the South Korean national air quality standards for annual average concentrations of PM_10_ and NO_2_ (50 μg/m^3^ and 30 ppb, respectively). The correlation coefficient between the two pollutants was 0.44.

**Table 2:** Summary statistics and Pearson correlation coefficient of individual-level PM_10_ and NO_2_ concentrations estimated at participant homes in the Korean CODA cohort (*n* = 457)

	Mean ± SD	IQR	Percentiles					Correlation coefficient(*r*)	
			5th	25th	50th	75th	95th	PM_10_	No_2_
PM_10_ (ug/m^3^)	43.1 ± 2.9	4.4	38.4	41.0	43.1	45.4	47.4	-	0.44^***^
NO_2_ (ppb)	13.6 ± 2.1	3.0	10.2	12.3	13.5	15.3	17.2		-

***: *p* < 0.0001

### Association between air pollution and lung function

Higher PM_10_ was significantly associated with lower FVC in all models; in our primary analysis adjusting for individual characteristics, a 4.4 μg/m^3^ IQR increase in PM_10_ concentration was associated with 0.13 L lower FVC (95% confidence interval (CI) = − 0.22 - -0.05, *p* = 0.003) (Table 3). The effect estimate for FEV_1_ was also negative but statistically non-significant in our primary model (regression coefficient = − 0.04, 95% CI = − 0.11 - 0.03, *p* = 0.29). Higher NO_2_ was significantly associated with lower FVC (regression coefficient = − 0.09, 95% CI = − 0.17 - -0.01, *p* = 0.035), while FEV_1_ was not associated with NO_2_ (Table 3).

**Table 3:** Effect estimates and 95% confidence intervals of FVC, FEV_1_, emphysema index, and mean wall area % for interquartile range increases in PM_10_ (4.4 μg/m^3^) and NO_2_ (3.0 ppb) in the CODA cohort

	All (*n* = 457)			
	PM_10_		NO_2_	
	*β* (95% CI)	*P*	*β* (95% CI)	*P*
FVC, L				
Model 1^a^	−0.13 (− 0.22, − 0.05)	0.003	− 0.09 (− 0.17, − 0.01)	0.035
Model 2^b^	− 0.13 (− 0.22, − 0.03)	0.011	− 0.10 (− 0.18, − 0.02)	0.017
Model 3^c^	− 0.12 (− 0.22, − 0.02)	0.015	− 0.09 (− 0.17, − 0.01)	0.029
FEV_1_, L				
Model 1^a^	− 0.04 (− 0.11, 0.03)	0.294	0.00 (− 0.06, 0.07)	0.881
Model 2^b^	− 0.02 (− 0.09, 0.06)	0.647	0.00 (− 0.07, 0.06)	0.950
Model 3^c^	−0.07 (− 0.14, 0.01)	0.078	− 0.01 (− 0.07, 0.05)	0.741
Emphysema index				
Model 1^a^	1.13 (0.25, 2.02)	0.012	0.35 (−0.48, 1.19)	0.406
Model 2^b^	1.08 (−0.08, 2.23)	0.068	0.35 (−0.49, 1.18)	0.412
Model 3^c^	1.13 (0.01, 2.25)	0.048	0.26 (−0.54, 1.07)	0.519
Mean wall area %				
Model 1^a^	1.04 (0.27, 1.80)	0.008	0.37 (−0.35, 1.10)	0.311
Model 2^b^	0.58 (−0.42, 1.58)	0.253	0.37 (−0.35, 1.09)	0.317
Model 3^c^	0.51 (−0.46, 1.49)	0.302	0.32 (−0.38, 1.02)	0.373

^a^Model 1 was adjusted for age, gender, education, height, weight, smoking, pack-years, medication use, and job

^b^Model 2 was adjusted for age, gender, education, height, weight, smoking, pack-years, medication use, job and calendar year at PFT test

^c^Model 3 was adjusted for age, gender, education, height, weight, smoking, pack-years, medication use, job, calendar year at PFT test, asthma and COPD

There were no significant interactions with the COPD status for the associations between either pollutant and lung function (Table 4). For PM_10,_ there was a significant interactions with smoking status for FVC with association only in ever smokers, (P interaction = 0.011, Table 5) and with sex with associations existing only in the larger group of men (*n* = 335) (P interaction = 0.021, Table 6). We found no interaction with overweight/obesity.

**Table 4:** Effect estimates and 95% confidence intervals of FVC, FEV1, emphysema index, and mean wall area % for interquartile range increases in PM_10_ (4.4 μg/m^3^) and NO_2_ (3.0 ppb) according to COPD status in the CODA cohort

	PM_10_				*P* for interaction	NO_2_				*P* for interaction
	Non-COPD (*n* = 161)		COPD (*n* = 296)			Non-COPD (*n* = 161)		COPD (*n* = 296)		
	*β* (95% CI)	*P*	*β* (95% CI)	*P*		*β* (95% CI)	*P*	*β* (95% CI)	*P*	
FVC, L										
Model 1^a^	−0.12 (−0.26, 0.03)	0.117	−0.13 (− 0.24, − 0.02)	0.018	0.900	−0.12 (− 0.25, 0.01)	0.071	−0.06 (− 0.15, 0.04)	0.261	0.436
Model 2^b^	−0.09 (− 0.25, 0.06)	0.226	− 0.11 (− 0.22, 0.00)	0.060	0.875	−0.15 (− 0.28, −.02)	0.024	−0.06 (− 0.16, 0.04)	0.231	0.256
Model 3^c^	−0.11 (− 0.26, 0.04)	0.161	− 0.13 (− 0.24, − 0.01)	0.032	0.865	− 0.15 (− 0.28, −.02)	0.023	−0.05 (− 0.15, 0.04)	0.268	0.229
FEV_1_, L										
Model 1^a^	− 0.09 (− 0.20, 0.02)	0.112	− 0.04 (− 0.12, 0.04)	0.359	0.451	−0.03 (− 0.13, 0.06)	0.515	0.00 (− 0.07, 0.08)	0.912	0.550
Model 2^b^	−0.09 (− 0.20, 0.03)	0.128	− 0.04 (− 0.12, 0.05)	0.387	0.452	−0.04 (− 0.14, 0.06)	0.419	0.00 (− 0.07, 0.08)	0.930	0.474
Model 3^c^	−0.10 (− 0.21, 0.02)	0.091	− 0.05 (− 0.13, 0.04)	0.270	0.456	−0.04 (− 0.14, 0.06)	0.413	0.01 (− 0.07, 0.08)	0.861	0.437
Emphysema index										
Model 1^a^	0.65 (−0.89, 2.19)	0.405	1.55 (0.52, 2.57)	0.003	0.337	−0.09 (−1.60, 1.38)	0.908	0.47 (− 0.48, 1.42)	0.332	0.523
Model 2^b^	0.24 (−1.50, 1.99)	0.789	1.21 (− 0.02, 2.44)	0.053	0.298	−0.23 (−1.70, 1.23)	0.756	0.51 (−0.43, 1.46)	0.288	0.392
Model 3^c^	0.41 (−1.30, 2.15)	0.641	1.39 (0.17, 2.62)	0.026	0.291	−0.22 (− 1.70, 1.23)	0.764	0.46 (− 0.48, 1.40)	0.337	0.429
Mean wall area %										
Model 1^a^	2.33 (1.00, 3.66)	0.001	0.64 (−0.25, 1.53)	0.159	0.037	0.33 (−0.95, 1.61)	0.614	0.34 (− 0.49, 1.17)	0.417	0.985
Model 2^b^	1.61 (0.10, 3.12)	0.037	0.05 (−1.00, 1.11)	0.922	0.055	0.16 (− 1.10, 1.42)	0.809	0.39 (−0.43, 1.21)	0.346	0.751
Model 3^c^	1.65 (0.13, 3.16)	0.033	0.09 (−0.97, 1.16)	0.862	0.055	0.16 (−1.10, 1.42)	0.807	0.38 (− 0.44, 1.20)	0.360	0.764

^a^Model 1 was adjusted for age, gender, education, height, weight, smoking, pack-years, medication use, and job

^b^Model 2 was adjusted for age, gender, education, height, weight, smoking, pack-years, medication use, job and calendar year at PFT test

^c^Model 3 was adjusted for age, gender, education, height, weight, smoking, pack-years, medication use, job, calendar year at PFT test, and asthma

**Table 5:** Effect estimates and 95% confidence intervals of FVC, FEV_1_, emphysema index, and mean wall area % for interquartile range increases in PM_10_ (4.4 μg/m^3^) and NO_2_ (3.0 ppb) according to smoking status in the CODA cohort

	PM_10_				*P* for interaction	NO_2_				*P* for interaction
	Never smoker (*n* = 165)		Ever (former/current) smoker (*n* = 292)			Never smoker (*n* = 165)		Ever (former/current) smoker (*n* = 292)		
	*β* (95% CI)	*P*	*β* (95% CI)	*P*		*β* (95% CI)	*P*	*β* (95% CI)	*P*	
FVC, L										
Model 1^a^	0.02 (−0.13, 0.16)	0.818	− 0.21 (− 0.32, − 0.10)	0.000	0.011	−0.05 (− 0.18, 0.07)	0.410	−0.11 (− 0.21, − 0.01)	0.038	0.510
Model 2^b^	0.02 (− 0.12, 0.17)	0.760	− 0.20 (− 0.32, − 0.09)	0.001	0.012	−0.07 (− 0.20, 0.05)	0.264	− 0.11 (− 0.21, − 0.01)	0.028	0.626
Model 3^c^	0.03 (− 0.12, 0.18)	0.683	− 0.20 (− 0.31, − 0.08)	0.001	0.010	− 0.07 (− 0.20, 0.06)	0.279	− 0.10 (− 0.20, 0.00)	0.049	0.708
FEV_1_, L										
Model 1^a^	0.04 (− 0.07, 0.16)	0.432	− 0.09 (− 0.17, 0.00)	0.046	0.064	0.00 (− 0.10, 0.10)	0.965	0.01 (− 0.07, 0.09)	0.825	0.861
Model 2^b^	0.06 (− 0.05, 0.18)	0.302	−0.07 (− 0.16, 0.02)	0.151	0.071	− 0.01 (− 0.12, 0.09)	0.770	0.01 (− 0.07, 0.08)	0.898	0.751
Model 3^c^	0.00 (−0.11, 0.11)	0.967	−0.10 (− 0.19, − 0.02)	0.020	0.133	−0.04 (− 0.14, 0.05)	0.408	0.01 (− 0.07, 0.08)	0.843	0.429
Emphysema index										
Model 1^a^	1.16 (−0.37, 2.68)	0.136	0.89 (− 0.18, 1.96)	0.103	0.773	0.20 (−1.20, 1.62)	0.781	0.42 (−0.61, 1.45)	0.421	0.800
Model 2^b^	1.08 (−0.57, 2.73)	0.200	0.79 (−0.56, 2.14)	0.252	0.755	0.19 (−1.20, 1.60)	0.794	0.42 (− 0.61, 1.45)	0.421	0.790
Model 3^c^	1.45 (−0.16, 3.06)	0.077	0.67 (−0.64, 1.98)	0.318	0.388	0.28 (−1.10, 1.65)	0.686	0.24 (−0.75, 1.24)	0.631	0.964
Mean wall area %										
Model 1^a^	0.75 (−0.55, 2.06)	0.257	1.20 (0.28, 2.11)	0.011	0.577	1.16 (− 0.05, 2.37)	0.061	−0.02 (− 0.90, 0.85)	0.956	0.114
Model 2^b^	0.39 (− 1.00, 1.80)	0.587	0.73 (−0.42, 1.88)	0.215	0.755	1.14 (−0.06, 2.34)	0.063	−0.03 (− 0.90, 0.84)	0.952	0.116
Model 3^c^	0.52 (−0.86, 1.90)	0.459	0.53 (−0.60, 1.65)	0.360	0.996	1.14 (−0.03, 2.31)	0.057	−0.10 (− 0.95, 0.75)	0.810	0.088

^a^Model 1 was adjusted for age, gender, education, height, weight, pack-years, medication use, and job

^b^Model 2 was adjusted for age, gender, education, height, weight, pack-years, medication use, job and calendar year at PFT test

^c^Model 3 was adjusted for age, gender, education, height, weight, pack-years, medication use, job, calendar year at PFT test, asthma and COPD

**Table 6:** Effect estimates and 95% confidence intervals of FVC and FEV_1_, emphysema index, and mean wall area % for interquartile range increases in PM_10_ (4.4 μg/m^3^) and NO_2_ (3.0 ppb) by gender in the CODA cohort

	PM_10_				*P* for interaction	NO_2_				*P* for interaction
	Male (*n* = 335)		Female (*n* = 122)			Male (*n* = 335)		Female (*n* = 122)		
	*β* (95% CI)	*P*	*β* (95% CI)	*P*		*β* (95% CI)	*P*	*β* (95% CI)	*P*	
FVC, L										
Model 1^a^	−0.20 (−0.30, − 0.10)	0.000	0.02 (− 0.14, 0.19)	0.762	0.021	−0.11 (− 0.20, − 0.02)	0.023	−0.03 (− 0.18, 0.12)	0.704	0.365
Model 2^b^	−0.19 (− 0.30, − 0.08)	0.001	0.03 (− 0.13, 0.19)	0.727	0.022	−0.12 (− 0.21, − 0.02)	0.015	−0.05 (− 0.20, 0.10)	0.527	0.436
Model 3^c^	−0.18 (− 0.29, − 0.07)	0.001	0.03 (− 0.13, 0.20)	0.697	0.024	−0.11 (− 0.20, − 0.02)	0.022	−0.04 (− 0.19, 0.11)	0.608	0.430
FEV_1_, L										
Model 1^a^	−0.07 (− 0.15, 0.01)	0.080	0.05 (− 0.08, 0.18)	0.423	0.103	−0.01 (− 0.08, 0.07)	0.856	0.03 (− 0.08, 0.15)	0.556	0.546
Model 2^b^	−0.05 (− 0.14, 0.03)	0.236	0.06 (− 0.06, 0.19)	0.325	0.122	−0.01 (− 0.08, 0.06)	0.756	0.02 (− 0.09, 0.14)	0.695	0.613
Model 3^c^	−0.09 (− 0.17, − 0.01)	0.034	0.00 (− 0.13, 0.12)	0.945	0.231	−0.01 (− 0.08, 0.06)	0.833	−0.02 (− 0.13, 0.09)	0.764	0.885
Emphysema index										
Model 1^a^	1.15 (0.14, 2.17)	0.026	1.08 (−0.64, 2.80)	0.219	0.942	0.42 (−0.54, 1.37)	0.391	0.15 (−1.50, 1.84)	0.861	0.786
Model 2^b^	1.09 (−0.21, 2.40)	0.100	1.04 (−0.77, 2.85)	0.261	0.957	0.43 (−0.52, 1.38)	0.374	0.08 (−1.60, 1.77)	0.922	0.723
Model 3^c^	0.95 (−0.31, 2.21)	0.139	1.54 (−0.22, 3.31)	0.086	0.549	0.26 (−0.67, 1.18)	0.586	0.29 (−1.30, 1.93)	0.724	0.968
Mean wall area %										
Model 1^a^	1.30 (0.42, 2.18)	0.004	0.25 (−1.20, 1.74)	0.744	0.227	0.19 (−0.64, 1.01)	0.657	0.98 (−0.49, 2.45)	0.190	0.352
Model 2^b^	0.86 (−0.27, 1.98)	0.135	−0.06 (−1.60, 1.51)	0.945	0.300	0.21 (−0.62, 1.03)	0.624	0.89 (−0.57, 2.34)	0.231	0.418
Model 3^c^	0.67 (−0.43, 1.77)	0.234	0.15 (−1.40, 1.69)	0.846	0.550	0.11 (−0.69, 0.91)	0.784	0.98 (−0.44, 2.40)	0.177	0.295

^a^Model 1 was adjusted for age, education, height, weight, smoking, pack-years, medication use, and job

^b^Model 2 was adjusted for age, education, height, weight, smoking, pack-years, medication use, job and calendar year at PFT test

^c^Model 3 was adjusted for age, education, height, weight, smoking, pack-years, medication use, job, calendar year at PFT test, asthma and COPD

### Association between air pollution and CT features

For CT features, both the emphysema index and wall area percent were significantly associated with PM_10_. For an IQR increase in PM_10_, the emphysema index increased by 1.13 (95% CI = 0.25–2.02, *p* = 0.012) and the wall area percent increased by 1.04 (95% CI = 0.27–1.80, *p* = 0.008, Table 3) in our primary model. However, there was no association between NO_2_ and the CT phenotypes. We repeated the analysis by including the calendar year of the pulmonary function measurement and history of asthma or COPD as a covariate and the associations for PM_10_ remained significant with the emphysema index, but not with the wall area% (Table 3). We also performed analysis on functional small airway disease and did not find any significant association (regression coefficient = 0.26, 95% CI = − 2.10 - 2.62, *p* = 0.83).

Stratified analysis by COPD status showed a stronger association of PM_10_ with the wall area percent among individuals without COPD (P interaction = 0.037, Table 4) There was no significant interaction with smoking status or gender (Tables 5 and 6).

## Discussion

In this study, we found that PM_10_ was associated with lung function, emphysema index, and wall area percent in the Korean CODA cohort. Higher long-term PM_10_ exposure was related to lower FVC and this association appeared to be limited to men or ever-smokers. We also found significantly different associations between PM_10_ and wall area percent by COPD status. There was significant association between NO_2_ and FVC. However, there was no association between NO_2_ and imaging phenotypes.

While most previous studies of long-term air pollution and lung function in older adults were based on general populations, the current study used a cohort including healthy subjects as well as a substantial proportion of COPD subjects and found that the association with FVC was also significant in the COPD subgroup. Increased ambient air pollution including PM_10_ and NO_2_ was associated with decreased lung function in healthy adults from the Study on Air Pollution And Lung Disease In Adults in Switzerland [33]. In middle-aged men and women from the Atherosclerosis Risk in Communities study in the United States, increased traffic-related air pollution was associated with decreased FEV_1_ and FVC [34]. In middle- to old-aged participants from the Framingham Heart study in the Northeastern United States, long-term exposure to traffic emission and PM_2.5_ was associated with decreased FEV_1_ as well as FEV_1_ decline [35]. In Japanese women, living in areas with a high level of air pollution was associated with large FEV_1_ decline [36]. In the National Emphysema Treatment Trial study, one of a few studies focusing on COPD patients, an increase in PM_2.5_ was associated with a rapid decline of FEV_1_ [37]. Our study suggests that the influence of PM air pollution could be larger for COPD patients than for the general population.

In the current study, a significant association of PM_10_ was observed with FVC, while no association was found with FEV_1_. Some studies reported the consistent patterns of stronger associations with FVC than FEV_1_, while others found the reverse pattern. A recent paper in UK reported higher effect estimates on FVC than FEV_1_ for PM_10_, but higher estimates on FEV_1_ for PM_2.5_ [9]. Whether PM is associated differently with lung volume or airflow limitation according to the size of the particles should be further investigated.

NO_2_ is an important marker of traffic-related air pollution and was associated with various endpoints including COPD in previous studies, although we did not find associations with imaging phenotypes. Our cohort of fewer than 500 participants might have not provided sufficient statistical power for detecting an association, although our results showed an association of PM_10_ with both lung function and CT measurements. Another possible explanation could be different features of pollution sources related to traffic between the two pollutants. With respect to traffic, PM results from re-suspended road dust generated by moving vehicles, tire and brake wear, and tailpipe exhaust, whereas NO_2_ is mainly emitted in vehicle exhaust. The low correlation coefficient between the two pollutant concentrations (0.44) also supports this explanation. The model performance for NO_2_ was better than for PM_10_, which can be explained by the large impact of local pollution sources on NO_2_ as opposed to PM_10_ affected by regional sources. The local sources are better characterized by geographic variables which are major input data of our prediction model. R^2^ values for PM_10_ are under 0.50 in other national models.

The effects of air pollution and lung function may vary by various factors such as gender, genetics, smoking status, diet, medication, and obesity. Modification by these factors is inconsistent according to the literature. In a previous general population study in Taiwan, the association between air pollution and lung function was stronger in females, the obese, and nonsmokers [38]. However, in the current study, we saw some evidence that men were more susceptible as found in previous studies, possibly because men are likely to spend more time outdoors [9, 39, 40]. However, our study had more men than women to begin with, and more male subjects smoked with a history of COPD, which may have affected our findings. Our results showed a significant association between PM_10_ and lung function in ever-smokers, but not in never smokers. This is consistent with the findings of the Framingham Heart study showing that former smokers are more susceptible to air pollution [35]. We did not find a significant interaction with overweight in the association with PM_10_, although there are reports that obesity is a risk factor for air pollution susceptibility. The modifying effects differ according to the population.

Recent studies have revealed that imaging features are associated with adverse clinical outcomes in COPD [11]. To our knowledge, this is the first study to investigate the association between air pollution and CT features in COPD subjects. There were at least three studies based on the general population. The Multi-Ethnic Study of Atherosclerosis (MESA) including 6515 participants showed only weak evidence of the association between PM and NOx and percent emphysema from cardiac CT scans [15]. The MESA study also showed significant associations between long-term exposure to air pollutants and emphysema progression [16]. Among 2545 nonsmoking Framingham CT sub-study participants, there was no evidence of the association between ambient air pollution and radiographic measures of emphysema or airway disease, whereas the odds of emphysema in former smokers increased for living near major roads [14]. In the current study, PM_10_ exposure was associated with increased emphysema index and wall area percent in participants with or without COPD. The depth of inspiration affects the results of the CT-derived airway measurements. An increase in the depth of inspiration results in a larger airway lumen and smaller airway thickness [41]. The influence of the inspiration level in the upper bronchus is significantly lower than that in the lower bronchus [42]. Therefore, airways were measured in the right apical and left apicoposterior segmental bronchi in our study to standardize the assessment of airway wall thickness, a measure of a chronic bronchitis phenotype. The association with wall thickness differed according to COPD status. PM_10_ exposure was associated with wall area percent especially in the non-COPD group. Occupational dust/fume exposure was associated with air trapping, and airway wall thickness in men [43] and our previous study of biomass exposure showed an association with wall area percent in smokers [44]. Our current results suggest that ambient air pollution can also influence airway thickening as well as worsen emphysema.

Our study has some limitations to address. First, we used modeled annul-average concentrations of air pollution at subjects’ home addresses at baseline as individual-level long-term exposure to air pollution, without incorporating early exposures in the life course. Household exposure and exposure varying by time-activities were not accounted for either. Future analyses considering highly-resolved exposure estimates with longitudinal address information and time activity data may address the impact of these limitations. We also used annual-average concentrations in the year of 2010 and applied to our cohort data started in 2012. We assumed that the spatial distribution of air pollution concentrations is consistent throughout the study period. Since this is a cohort study which relies on the spatial contrast of air pollution across participants, a change of concentrations over 5 years may not matter as much compared to the change in spatial ranking of high and low pollution areas. Our previous study showed high correlation (Pearson correlation coefficient = 0.94) between 4-year averages for 2009–2012 and annual averages in 2010 across about 300 air quality regulatory monitoring sites [45]. Annual average concentration of PM_10_ and NO_2_ were below the South Korean national air quality standard (50 μg/m^3^ and 30 ppb, respectively). However, these are still higher than the average concentrations and the air quality standards in the US and Europe where many studies reported the associations with respiratory outcomes. Secondly, as some previous epidemiological studies reported, PM_2.5_ may be strongly associated with COPD compared to PM_10_ or NO_2_. It is not feasible to include PM_2.5_ to this study because national-scale PM_2.5_ regulatory monitoring data are available since 2015. The sample size is relatively small. However, our strength using standardized spirometry and quantitative CT measurement using a single CT scanner could have allowed us to detect the association. This cohort recruited participants near cement plants, generalizability to areas without such point source may be reduced.

## Conclusions

In conclusion, both lung function and imaging phenotypes (emphysema and airway wall thickening) were associated with PM_10_ exposure in this population of older adults. We found evidence of differences in associations by sex, smoking and COPD status.

## Data Availability

The datasets used and/or analysed during the current study are available from the corresponding author on reasonable request.

## Abbreviations

BMI: Body mass index
CODA cohort: Chronic obstructive pulmonary disease in dust areas cohort
COPD: Chronic obstructive pulmonary disease
CT: Computed tomography
FEV_1_: Forced expiratory volume in 1 s
FVC: Forced vital capacity
PM: Particulate matter
HU: Hounsfield unit
